# Randomized Clinical Trial of Antioxidant Therapy Patients with Septic Shock and Organ Dysfunction in the ICU: SOFA Score Reduction by Improvement of the Enzymatic and Non-Enzymatic Antioxidant System

**DOI:** 10.3390/cells12091330

**Published:** 2023-05-06

**Authors:** Alfredo Aisa-Álvarez, Israel Pérez-Torres, Verónica Guarner-Lans, Linaloe Manzano-Pech, Randall Cruz-Soto, Ricardo Márquez-Velasco, Sergio Casarez-Alvarado, Juvenal Franco-Granillo, Marcela Elizabeth Núñez-Martínez, María Elena Soto

**Affiliations:** 1 Critical Care Department, American British Cowdray (ABC) Medical Center, I.A.P. ABC Sur 136 No. 116 Col. Las Américas, México City 01120, Mexico; 2 UNAM Master’s and Doctoral Program in Medical, Dental and Health Sciences UNAM, México. Av. Universidad 3000, Coyoacán, México City 04510, Mexico; 3 Cardiovascular Biomedicine Department, Instituto Nacional de Cardiología Ignacio Chávez, Juan Badiano 1, Sección XVI, Tlalpan, México City 14080, Mexico; 4 Physiology Department, Instituto Nacional de Cardiología Ignacio Chávez, Juan Badiano 1, Sección XVI, Tlalpan, México City 14080, Mexico; 5 Immunology Department, Instituto Nacional de Cardiología Ignacio Chávez, Juan Badiano 1, Sección XVI, Tlalpan, México City 14080, Mexico; 6 Central Laboratory Department, ABC Hospital Observatory, Sur 136 No. 116 Col. Las Américas, México City 01120, Mexico; 7 Department of the Cardiovascular, Division of the American British Cowdray Medical Center, Sur 136 No. 116 Col. Las Américas, México City 01120, Mexico

**Keywords:** multiple organ failure, antioxidants, Vitamin C, Vitamin E, N-acetylcysteine, melatonin

## Abstract

Background and aim: Here, we assess the effect of adjuvant antioxidant therapies in septic shock patients with organ dysfunction and their effect on the enzymatic and non-enzymatic antioxidant systems. Methods: Randomized clinical trial run between 2018 and 2022. One hundred and thirty-one patients with septic shock were included in five groups with 25, 27, 24, 26 and 29 patients each. Group 1 received vitamin C (Vit C), Group 2 vitamin E (Vit E), Group 3 n-acetylcysteine (NAC), Group 4 melatonin (MT) and group 5 no treatment. All antioxidants were administered orally or through a nasogastric tube for 5 days as an adjuvant to standard therapy. Results: All patients had multiple organ failure (MOF) and low Vit C levels. Vit C therapy decreased CRP, PCT and NO_3_^−^/NO_2_^–^ but increased Vit C levels. The SOFA score decreased with MT in 75%, Vit C 63% and NAC 50% vs. controls 33% (*p* = 0.0001, *p* = 0.03 and *p* = 0.001 respectively). MT diminished lipid peroxidation (LPO) (*p* = 0.01) and improved total antioxidant capacity (TAC) (*p* = 0.04). Vit E increased thiol levels (*p* = 0.02) and tended to decrease LPO (*p* = 0.06). Selenium levels were decreased in the control group (*p* = 0.04). Conclusions: Antioxidants used as an adjuvant therapy in the standard treatment of septic shock decrease MOF and oxidative stress markers. They increase the TAC and thiols, and maintain selenium levels.

## 1. Introduction

Sepsis and septic shock cause a high mortality in Intensive Care Units (ICU) throughout the world and 80% of patients with this condition are admitted with multiple organ failure (MOF) [1]. The prevalence of sepsis and septic shock varies in different countries, being three cases/1000 inhabitants in the United States, of which 51% are managed in the ICU and 17.3% in the intermediate care units or coronary care (IUCC) [2]. In a multicenter study conducted in México in 68 emergency medical services, of which five were private hospitals and 63 public hospitals and where a total of 2379 patients were analyzed, it was found that 307 subjects presented sepsis and 41 septic shock. The global prevalence of sepsis and septic shock was 12.9% and 13.3%, respectively, and a global mortality of 16.93% was reported. In a separate evaluation, 9.3% of deaths were due to sepsis and 65.85% to septic shock. Therefore, the authors concluded there is an association between the presence of septic shock and mortality [3].

These conditions generate high costs to the public health system worldwide and to the families of the patients. Therefore, different strategies have been evaluated to predict the prognosis and reduce costs [4]. At the same time, new therapies have been proposed that focus on the pathophysiology of these conditions [5,6,7,8] which involves inflammation [9], epigenetic status [10], metabolism [11] and alterations of the microbiota [12,13].

However, there are other mechanisms involved during sepsis including deregulation of hemodynamic and oxidative stresses (OS), which exert a synergistic effect with inflammation and lead to dysfunction in various organs. Therefore, the use of antioxidants is currently being investigated [14,15,16].

The systemic response that triggers sepsis leads to dysfunction in the heart through compensatory reaction of the sympathetic nervous system, leading to micro vascular dysfunction with activation of the immune system. The activation of the immune system damages tissues through a cascade that involves damage-associated molecular patterns, pathogen-associated molecular patterns [17], the complement system, cytokine release and the presence of inflammatory molecules such as TNFα, which is associated with an increase in OS [18,19,20,21]. In this state of oxidative deregulation, pro-oxidant molecules that originate from the superoxide anion (O_2_^–^), such as hydroxyl radical (OH), hydrogen peroxide (H_2_O_2_) and peroxynitrite (ONOO^–^) among others, can interact and favor the damage to proteins, enzymes, cell membrane lipids and DNA. Damage to these molecules contributes to damage processes in different cell populations which result in apoptosis and an increase in autophagy, leading to cell and tissue death and subsequent MOF [22]. The inflammatory response in sepsis also plays a fundamental role and therefore, standard therapy includes the use of steroidal and non-steroidal anti-inflammatory drugs that have led to very limited results [23]. This can be due to the variability in the response of each individual and to the large number of molecular pathways that interact in this condition and may explain the prognosis [24,25].

On the other hand, antioxidant therapy in septic shock has been proposed since Hippocrates, who used myrrh (*Commiphora mukul*, *Commiphora myrrha*) [26] for therapeutic and anti-inflammatory medicinal purposes [27]. Currently, the use of antioxidant therapy in patients with septic shock has shown its usefulness in reducing OS markers [28,29,30,31]. Similarly, we found that antioxidant therapy added to standard management in septic shock increases total antioxidant capacity (TAC) and decreases OS and MOF in a previous study by our group [32]. Moreover, we demonstrated this same clinical effect with the use of the antioxidant therapy in patients in severe condition due to SARS-CoV-2 infection during the pandemic [33]. The results confirm previous findings on the usefulness of the antioxidant therapy in patients with severe septic shock and organ damage [28,29,30,31,34].

Therefore, the aim of this study was to evaluate the use of antioxidants concomitantly with standard therapy in patients with septic shock with MOF, through a randomized clinical trial (RCT). We also evaluate the effect on various biomarkers of the enzymatic and non-enzymatic antioxidant system, before and after therapeutic intervention.

## 2. Materials and Methods

### 2.1. Population Study

This was a randomized and blinded longitudinal prospective clinical trial in a cohort of patients that was run between April 2018 and January 2022. The population studied included patients older than 18 years of any gender who were admitted to the intensive care unit of the ABC Medical Center, Observatory and Santa Fe campus with a diagnosis of septic shock [34]. Patients were included within 24 h after admission after giving their informed consent. Patients with an advance directive form, previous chronic or recent use of steroids, statins or antioxidants, patients with reported allergies to antioxidants or with contraindication to the use of Vitamin C (Vit C), Vitamin E, (Vit E), N-Acetylcysteine (NAC) or melatonin (MT), and pregnant women were excluded. Clinical and laboratory variables were assessed and the measurement of the sequential organ failure assessment score (SOFA) was done every day until discharge from the ICU.

### 2.2. Sample Size

The sample size was calculated considering the difference in the means of low ascorbic acid levels and improvement with the treatment using antioxidants. It suggested the inclusion of 11 patients in each group for a desired 80% power and an alpha error of *p* < 0.05 based on a previous reference [35].

### 2.3. Ethical Aspects

A signed informed consent form was obtained from each participant as recommended in the Declaration of Helsinki, modified in the Tokyo Congress, Japan. The research was approved by the Ethical, Biosecurity and Investigation Committees of the National Institute of Cardiology (registration number INCICh: PT 10-0-76) and Centro Medico ABC Campus Observatory number ABC-18-19, Trial Registration: ClinicalTrials.gov Identifier: NCT 03557229.

### 2.4. Randomization

**Figure 1:**
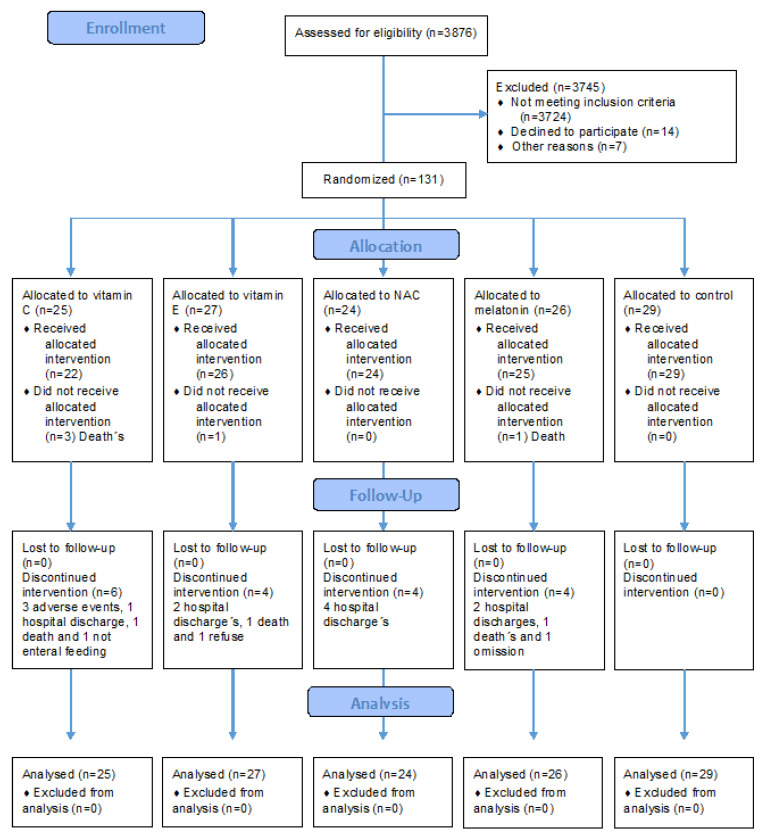
Electronic selection by computer was used to divide patients into blocks with a total of 5 groups of 131 patients in total. In the group Vit C (n = 27) patients were studied, in the group with Vit E (n = 24), NAC (n = 24), MT (n = 26) and (n = 29) patients remained without treatment.

Electronic selection by computer was used to divide patients into blocks with a total of 5 groups with around 25 patients in each one. Group 1 received Vit C, group 2 Vit E, group 3 NAC and group 4 MT; group 5 patients remained without treatment (Tx) and formed the control group. Personnel unrelated to the study participated in the blinding and placed the indicated therapy in identical opaque envelopes numbered from 1 to 125 and these were applied consecutively. The randomized process is shown in Figure 1.

### 2.5. Data Collection

A medical examination and a complete clinical history were performed on each patient upon admission to the ICU, and the prognostic scales of APACHE II [36] and SAPS II were calculated for 7 days [37]. Pre- and post-treatment samples were taken to analyze complete blood count, blood chemistry, electrolytes, liver function tests, C-reactive protein (CRP), procalcitonin (PCT), venous and arterial blood gases and OS markers.

### 2.6. Description of the Intervention

In addition to the standard therapy, each group of patients received an antioxidant orally or by nasoenteral tube for 5 days. In the NAC group, 600 mg effervescent tablets were administered every 12 h; in the MT group, extended-release capsules of 50 mg in a daily dose were administered; in the Vit C group, 1 g tablets every 6 h and in the Vit E group, capsules α-tocopherol of 400 IU were given every 8 h.

### 2.7. Sample Collection and Storage

A quantity of 20 mL of blood was obtained upon admission and 48 h after treatment. Samples were identified as pre (0 h) or post sample (48 h). They were centrifuged at 3000 rpm for 20 min at 4 °C. Serum was stored in 3 or 4 Eppendorff aliquots of 1.5 mL and stored at <70° until processed.

### 2.8. Evaluation of the Antioxidant Enzymes

#### 2.8.1. GPx Activity

A quantity of 100 μL of serum was suspended in 1.6 mL of 50 mM phosphate buffer (KH_2_PO_4_, pH 7.3), 0.2 mM NADPH, 1 mM GSH, and 1 IU/mL glutathione reductase. The mixture was incubated for 3 min at 37 °C, then 100 μL of 0.25 mM H_2_O_2_ were added to start the reaction and the absorbance was monitored for 7 min at 340 nm [38]. Activity is expressed in μmol NADPH oxidized/min/mL in serum with an extinction coefficient of 6220 M^−1^ cm^−1^ at 340 nm of NADPH.

#### 2.8.2. GST Activity

A quantity of 100 μL serum was added to 700 μL phosphate buffer (KH_2_PO_4_, 0.1 M, pH 6.5) with 100 μL 0.1 mM GSH and 100 μL of 0.1 mM 1-chloro-2,4-dinitrobenzene (CDNB). The sample was incubated and monitored for 7 min at 37 °C and read at 340 nm. GST activity was expressed in units of GS-DNB μmo/min/mL of serum with an extinction coefficient of 14,150 M^−1^ cm^−1^ [39].

#### 2.8.3. TrxR Activity

TrxR activity was assessed as previously described [40]. A quantity of 100 µL of serum was suspended in 3 mL of 0.1 mM phosphate buffer (KH_2_PO_4_, pH 7.0). NADPH 0.2 mM, EDTA 1 mM and 0.1 mg/mL bovine serum albumin free of fatty acids were added. The sample was read in the presence of 20 μL of the TrxR-specific inhibitor (10 μM auranofin), and together with a duplicate of the sample without the inhibitor. DTNB oxidation was monitored at 412 nm at 37 °C for 6 min with an extinction coefficient of 13,600 M^−1^ cm^−1^.

#### 2.8.4. Extracellular Super Oxide Dismutase (ecSOD) Activity

ecSOD activity was determined by electrophoresis in native 10% polyacrylamide gels. Electrophoresis was carried out at 120 V for 4 h, as previously described by Pérez-Torres et al. [41]. In brief, 100 μL of serum were used; the gel was incubated in 2.45 mM NBT solution for 20 min. The liquid was discarded and later it was incubated in a TEMED solution with 36 mM potassium phosphate (pH 7.8) and 0.028 mM riboflavin. The gel was exposed to a UV light lamp for 10 min and washed with distilled water to stop the reaction. A standardized curve was obtained with a serial dilution (2.5, 5, 10, 15, 30, and 60 ng) with SOD from bovine erythrocytes (Sigma Aldrich Chemical S.A. de R.L. de C.V. Toluca, México). SOD activity was calculated.

#### 2.8.5. Peroxidase Activity

Measurement of peroxidase activity was carried out by electrophoresis in native 10% polyacrylamide gels as previously described by Pérez-Torres et al. [41]. In brief, 100 μL of serum and 35 μL horseradish peroxidase (178.5 μg/mL) were loaded as a standard and added to a 10% polyacrylamide gel. To observe the peroxidase activity, the gel was washed with water three times for 5 min, then it was incubated with a mixture of 3 µg/mL of 3,3,5,5-tetramethylbenzidine dissolved in a solution of ethanol: acetic acid: water (1:1:1 vol/vol) with H_2_O_2_ for 10 min in the dark. Gels for peroxidase activity were analyzed by densitometry with a Kodak Image^®^ 3.5 system, and the activities were calculated following the technique described for ecSOD.

#### 2.8.6. GR Activity

For GR activity, 100 μL of serum were utilized according to the previously described method [42]. The GR activity is expressed as μmol of reduced GSSG/min/mg protein with an extinction coefficient of 6220 M^−1^ cm^−1^, and the absorbance was read at 340 nm.

### 2.9. Oxidative Stress Markers

#### 2.9.1. Determination of Selenium (Se)

Selenium (Se) determination was performed using 200 µL of serum according to the method described by Soto et al. and the absorbance was read at 600 nm [43].

#### 2.9.2. Thiols

For the measurement of thiols, to 25 mL of serum 100 μL KBH_4_ solution (1 mg/mL concentration) were added. The sample was mixed in a vortex for 15 sec and incubated for 3 min., after which 100 μL of buffer (formaldehyde solution 0.1 mL in 100 mL tridistilled water plus 3.38 mg EDTA and 1.21 g 100 mM Tris Base, pH 8.2), were added and mixed with a vortex for 15 s. The sample was incubated for 3 min. Subsequently, 100 μL 5, 5′ Ditiobis-2-nitrobenzoic acid (DTNB) solution were added (4 mg/mL), vortexed for 15 s and incubated for 3 min. For the reading, a growing curve was made with oxidized glutathione (GSSG) at 0.5, 10, 20, 40, 80, 160 μg and read at 415 nm.

#### 2.9.3. Total Antioxidant Capacity (TAC)

A quantity of 100 μL of serum was used for the TAC determination. The absorbance was measured at 593 nm, according to the method described by Benzie and Strain [44].

#### 2.9.4. Lipid Peroxidation (LPO)

A quantity of 100 μL of serum was used to determine LPO products, making them react with thiobarbituric acid as previously reported and measuring the absorbance at 532 nm [43].

#### 2.9.5. NO_3_^−^/NO_2_^−^ Ratio

The method reported by Griess was used for the determination of NO_3_^−^/NO_2_^−^ ratio. A quantity of 100 µL of the serum was incubated with 5 units of nitrate reductase plus NADPH, and the absorbance at 540 nm was measured.

#### 2.9.6. Carbonylation

A quantity of 100 μL of serum was used and protein carbonylation was detected spectrophotometrically as previously described [45]. Absorbance was read in a spectrophotometer at 370 nm, using water bidistilled as blank and a molar absorption coefficient of 22,000 M^−1^ cm^−1^.

### 2.10. Statistical Analysis

Continuous variables were expressed as mean ± standard deviation or median with minimum and maximum ranges. Categorical variables such as frequencies and percentages were also reported. Normality distribution was evaluated by Shapiro–France. For the graphic analysis of the distribution of the variables, histograms and/or stems of leaves graphics were employed. To test the significance of the results, we used nonparametric (Mann–Whitney) or Student’s t tests for independent measurements. Paired t with Friedman, Wilcoxon signed rank test or Kruskal–Wallis t test were used according to the number of comparisons in groups of two or multiples and according to Gaussian distribution. In some variables, standardization was made and in multiple comparisons, adjustment was made by Bonferroni correction. For the paired analysis (before-after), we used the Friedman or Wilcoxon tests with signed rank test according to the distribution of the data. For the comparison of proportions for two groups, Pearson’s Chi-square χ^2^ or Fisher’s exact test were employed. For the multivariate analysis, we used binary logistic regression, analysis of repeated samples and panel data testing of different models (grouped model, model for longitudinal data, marginal approximation model and multilevel model). For the survival analysis, we performed life tables and the method of Kaplan–Meier. Differences were considered as statistically significant when the *p* value was <0.05. Statistical analyses were performed using STATA V.16 Software and Sigma Software Plot 14 program (Jendel Corporation, 1986–2017).

## 3. Results

The demographic characteristics according to the assigned group are shown in Table 1 and Table 2. A total of 131 patients were included; 61 (47%) were men and 70 (53%) were women with a median age of 68 (58–78). Groups were comparable to each other since the presence of comorbidities, organic dysfunction scores and severity were similar between groups, and they showed no statistical difference. The differences with statistical significance between the groups were (1) the basal levels of lactate which were higher among patients who would receive Vit E, MT and the control group versus the groups with Vit C and NAC, and (2) the basal levels of platelets in patients who would receive Vit E and NAC, which were lower versus the other groups. However, it is worth mentioning that all the levels of platelets between the groups were similar since none had abnormalities according to the normal ranges of the laboratory.

**Figure 2:**
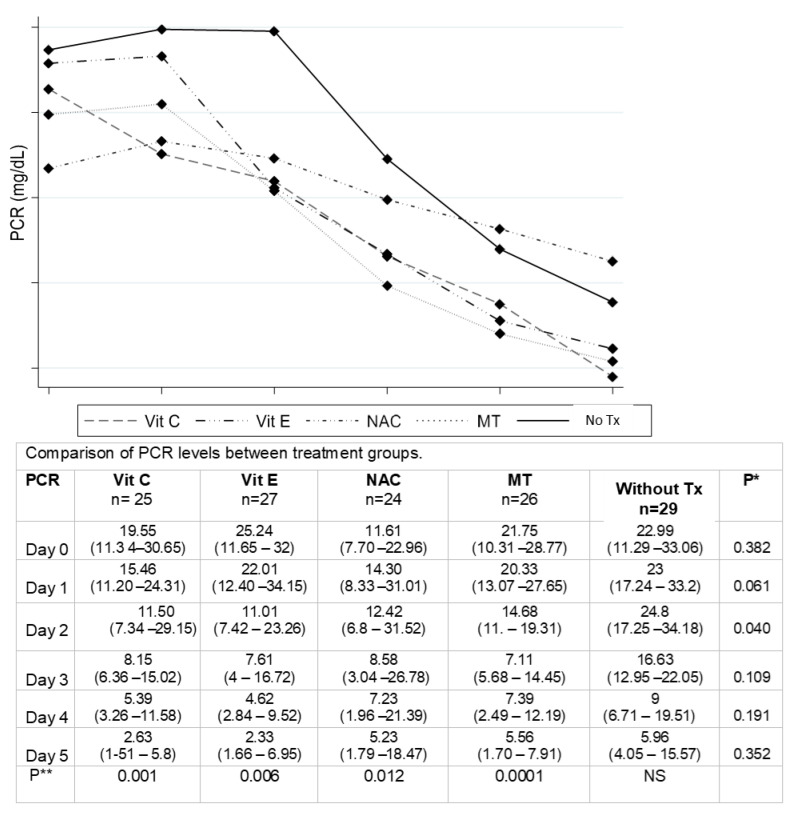
PCR = C-reactive protein; Vit C = vitamin C; Vit E = vitamin E; NAC = n-acetylcysteine; MT = melatonin; Tx = treatment, Values median. Test statistician: Kruskal-Wallis. p* Values are expressed as median (p25–p75). The variables were transformed to the normal by natural log and inverse logarithm. The evaluation over time was carried out using the repeated measures test p**.

Figure 2 shows that there was an evident decrease in the CRP levels with the application of all the antioxidant therapies, which was statistically significant when compared to the untreated control group and through the repeated analysis over time.

**Figure 3:**
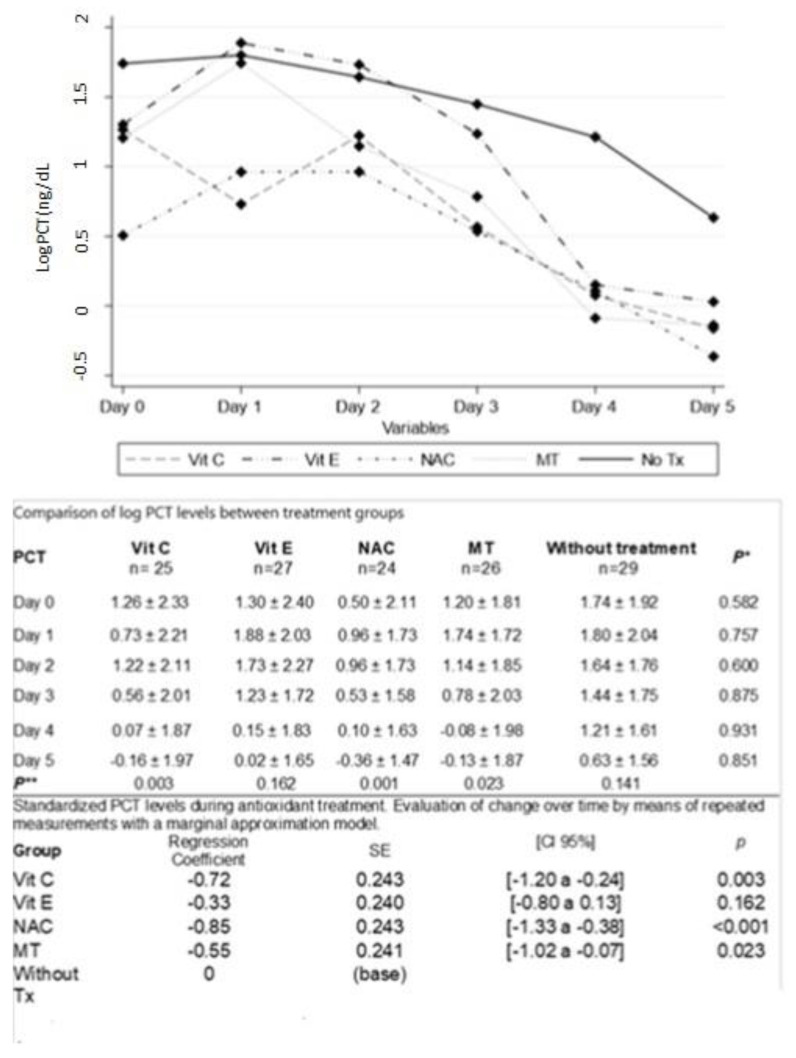
PCT = procalcitonin; Vit C = vitamin C; Vit E = vitamin E; NAC = n-acetylcysteine; MT = melatonin; Values are expressed as mean ± SD. Test Statistic: One-Way Kruskall Wallis between groups p* without difference but in the Repeated measures analysis. We Showed the progressive decrease in PCT levels with statistical significance in treatment groups Vit C, NAC and MT p** α ≥ 0.01. Without important decreased in groups Vit E and in the group without treatment, both had levels higher since basal status.

Figure 3 shows decreased PCT from the first days in the group treated with Vit C. In the group treated with NAC and MT, PCT levels decreased on the third day. With Vit E, it decreased on day 4. The differences were statistically significant when all groups were compared with the group without treatment whose decrease was less and was only observed until day 5.

**Figure 4:**
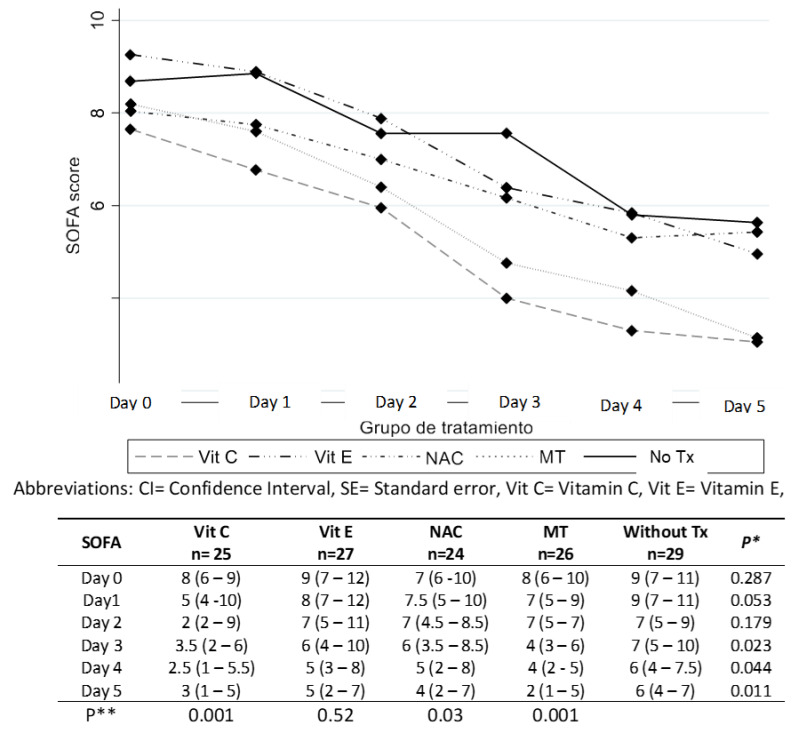
NAC = N-acetylcysteine, MT = Melatonin, Tx = without treatment, SOFA = Sequential organ failure assessment. Values are expressed as median (p25–p75 quartiles), Test statistic: One-way Kruskall Wallis p* and Repeated measures analysis of SOFA score p** changes over time relative to each treatment group: marginal approximation model.

Figure 4 shows a decrease in organ damage measured by the SOFA score. All the patients showed high scores only on the first day or day 0 without a difference between them. On day 1, there is a tendency to decrease the score with all antioxidants. On day 3, the decrease in the score continued in all patients. However, in the group of patients that received Vit C, there was a decrease in the value comparable to the baseline score, from 8 to 3.5 (56%), and this effect showed significant difference when compared with the other groups. During the 5 days that the patients received therapy, the group that received Vit C had a diminished score from 8 to 3 (63%), the group with Vit E from 9 to 5 (44%), the group with NAC from 7 to 4 (43%) and the group with MT from 8 to 2 (75%). The decrease in the group without treatment was from 9 to 6 (33%). The highest percentages of reduction were obtained with Vit C and analysis showed a decrease in the SOFA score in relation to the time of treatment with statistically significant difference in the groups treated with Vit C, Vit E NAC and MT. The difference between the treated and untreated groups was obtained by repeated measures statistical analysis.

**Figure 5:**
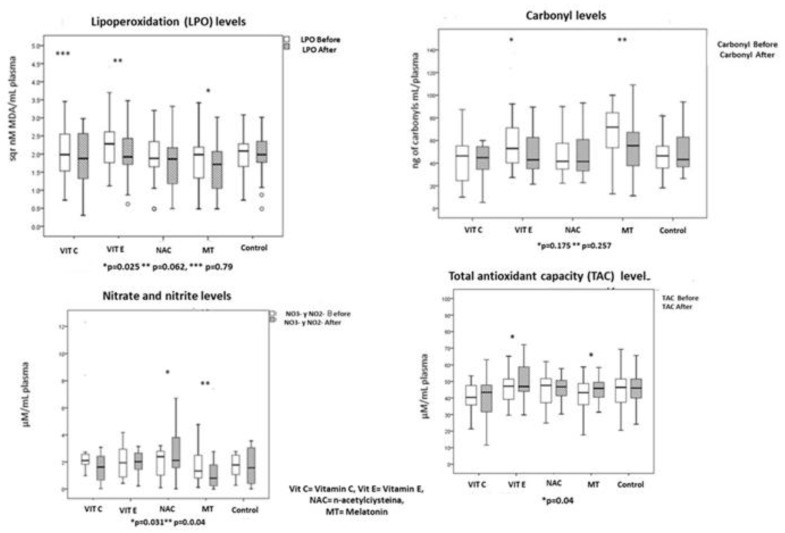
Showed oxidative stress markers in plasma of patients with sepsis before and after of antioxidant therapy.

**Figure 6:**
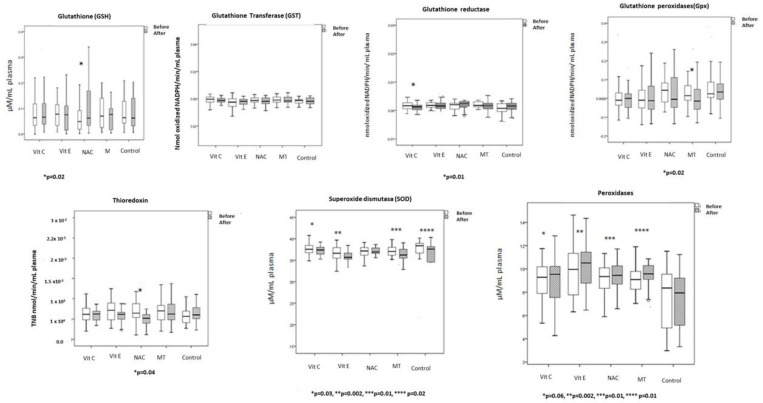
Enzymatic pathway that comprised the activity of the glutathione-S-transferase, glutathione reductase, glutathione peroxidase, Thioredoxin, superoxide dismutase, peroxidases and glutathione before and after antioxidant therapy. Abbreviations: Vit C = Vitamin C, Vit E = Vitamin E, NAC = n-acetyl cisteine, MT = Melatonin.

**Figure 7:**
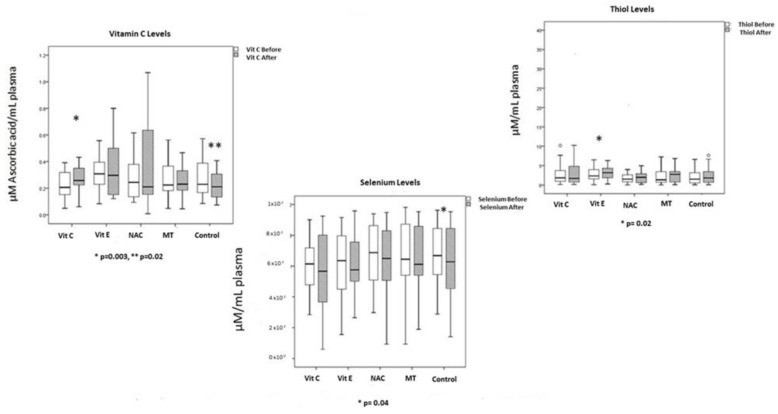
Showed some markers of the non-enzymatic pathway such as Vitamin C, Thiols and selenium levels before and after antioxidant therapy.

The OS markers and the enzymatic and non-enzymatic activities are shown in Figure 5, Figure 6 and Figure 7. The basal LPO was elevated in all the patients. Levels decreased in all groups; however, there were differences when compared between groups. LPO decreased six to nine times in the groups treated with Vit C and Vit E; in comparison with the control and MT groups, the difference between groups also reached a statistical significance (*p* = 0.02).

There was a six-fold decrease in carbonylation levels in the groups treated with Vit E, NAC and MT. However, in the groups treated with Vit C and without treatment, the carbonylation levels did not decrease. The enzymatic and non-enzymatic pathway is shown in Figure 6 and Figure 7. Regarding adverse effects of the antioxidant treatments, only one patient who received Vit C had abdominal pain and another had a skin rash. One patient who received MT had drowsiness. No adverse effects were reported in patients with NAC and Vit E.

## 4. Discussion

The participation of OS in septic shock and MOF has been previously reported [46,47]. The adaptation of the organism in the initial phases of OS, to balance the overproduction of reactive species (ROS) through processes of genetic overexpression and enzymatic activation, is crucial when the antioxidant systems are overcome. If the TAC is depleted, the damage to tissues and organs varies in intensity and duration. In this adaptation phase, partial or total protection can be obtained against organ damage with the use of antioxidants [4,5].

In this study, we found that patients with septic shock had a high SOFA score on admission to the ICU. They had high PCT and CRP levels, elevated LPO and carbonylation, and decreased TAC. These variables were compensated by the treatment with antioxidants.

The patients who received treatment with vitamin C and vitamin E had a decrease in LPO levels and although the difference did not reach a statistical significance, we can interpret that there is a tendency of six to seven times greater decrease with the use of this therapy. Our findings resemble those previously reported in an animal model where Vit C decreased LPO [48]. In these preliminary findings of treatment in humans, we support that the early use of Vit C and E can attenuate the alterations caused by OS in MOF [49]. However, reproducibility in humans to reach statistical significance may require a larger number of samples [4,5].

Vit C is an enzymatic cofactor with an antioxidant function derived from its ability to act as an electron donor. It reduces LPO and carbonylation, O_2_^–^, H_2_O_2_ and hypochlorite ion levels, and maintains GSH and Vit E levels. It also elevates peroxidases such as myeloperoxidase, which is reduced in sepsis [50]. Our results showed that the activities of peroxidases were increased with all treatments. This result is very important because the activity of the innate immune system is depleted in MOF. Our results suggest that, independently of antioxidant selected, the treatment can favor the activities of these enzymes which contribute to strengthening the immunologic system. In addition, Vit C inhibits the expression of mRNA for the inducible nitric oxide synthase (iNOS), which leads to overproduction of nitric oxide (NO) and is overexpressed in MOF. By inhibiting this enzyme, it prevents the abundant production of OONO^–^ and the presence of ROS. Large quantities of ONOO^–^ are produced during septic shock, and they have deleterious effects on different tissues, especially blood vessels, causing vasodilation which contributes to hypotension in MOF [51].

In addition, LPO was blocked by Vit E treatment in our series of patients. This vitamin binds to the cell membrane and decreases the polyunsaturated fatty acid oxidation due to its lipophilic characteristics. The oxidation of these fatty acids is increased in proinflammatory states. Therefore, supplementation with this vitamin in patients with sepsis may modulate the excessive inflammatory response coordinated by macrophages [52]. The effects of Vit E in our results could be explained by the activity and the persistent recycling of oxidized Vit E from Vit C, since Vit E is regenerated from ascorbic acid. In addition, its antioxidant activity in serum may be determined by the diminution of plasmatic levels of Vit C. The uncompensated decrease in Vit C was associated with greater severity in patients [53,54].

Protein oxidation can be estimated by carbonylation, which results from the direct oxidation of amino acid side chains and from oxidative cleavage of protein. Carbonyls are difficult to induce and therefore may indicate a more severe state of OS [55,56]. In this study, there was a tendency of carbonylation to be reduced in patients who were treated with Vit E. This suggests that the low levels of Vit C found in patients requires its supplementation in combination with Vit E to reduce carbonyls [57].

At present, there are still controversies regarding the complementary use of Vit C in the general therapy of patients with septic shock. In a systematic review with a meta-analysis that included 16 randomized clinical trials, it was concluded that Vit C does not help improve clinical outcomes in patients [58]. However, it is important to mention that the aim of these authors was to determine mortality as the outcome, and they did not evaluate MOF as an outcome. In contrast, to determine MOF was the main objective in our study. A limitation of the review was that the doses of Vit C employed in the different studies varied significantly and that the secondary results were obtained from a small number of randomized clinical trials (RCTs) with high heterogeneity. Although they used the random effects model, the results were not conclusive to support the use of Vit C. However, the authors justified the need to carry out RCTs due to the limitations of their study. In our study, mortality was not different between the group treated with the antioxidants and the non-treated group. Nevertheless, we cannot be conclusive in relation to this outcome because the sample size of the included population was not calculated for this purpose.

In the group treated with Vit C, the levels of ecSOD were decreased. This effect may be due to the fact that this enzyme regulates the O_2_^–^ and prevents its transformation into H_2_O_2_ which may be part of the regulatory adaptive phase to OS. On the other hand, our results showed a reduction in the GR activity. This enzyme participates in the regeneration of the GSH which is used in part by GPx for the elimination of H_2_O_2_ and also allows for the maintenance of the concentration of GSH. In addition, GSH is useful in the recovery of Vit C and Vit E after participating in the elimination of the ROS generated in situ or remotely. The decrease in the activity of GR may lead to a decrease in the GSH concentrations and in turn, this could drive an increase in the levels of ROS. Our results showed that the activity of GR was decreased in the group treated with Vit C. In addition, another enzyme that employed the GHS in the detoxification process is the GST. This enzyme is crucial in the detoxification of xenobiotics. Our results showed that the GST activity remained unchanged; however, in the group without treatment it was decreased, suggesting that it may favor its activity contributing to decreased LPO indexes and MOF independently of the antioxidant treatment selected.

Regarding the findings associated with the clinical part, the use of Vit C did not decrease the number of days of hospital stay, but its use was related to a lower percentage of the employment of inotropes and invasive mechanical ventilation, which was observed in all patients that received antioxidant therapy. In the treated groups, the percentage of inotropic use was 15.6% and mechanical ventilation was necessary in 11%, while in the untreated groups, it was of 31% and 19%, respectively. However, the difference did not reach a statistical significance.

A more recent meta-analysis reported that Vit C showed no evident clinical improvement and therefore effectiveness of the therapy was not recommended [59]. It is important to mention that in some studies, the basal levels of Vit C have been found to be below the reference value in patients with septic shock [60]. In this study, the patients had decreased levels on admission. In patients with septic shock, hypovitaminosis has been reported. This condition presents acutely in patients with sepsis and is secondary to metabolic consumption since intestinal absorption is not affected [61].

One of the main objectives to be evaluated in this study was whether the use of antioxidants decreased organ damage. We found that with the early use of Vit C, patients had a decrease in organ damage measured through the SOFA score and that this occurred since the beginning (first day of therapy with this antioxidant), showing a 37.5% reduction. On the fifth day, the decrease reached 63%. This finding confirmed the effects seen in a previous study, in which a decrease in the SOFA score in patients who received MT and Vit C was observed in a smaller number of patients [32].

In this study, this same effect was achieved with the use of NAC, which also reduces mortality [62]. NAC has been applied in animal models where it reduced the organ damage induced by shock caused by endotoxin. NAC showed effects when given before and after endotoxin and it reduced the ROS [63]. In the therapy already applied in humans, it improves the TAC [32], and we have confirmed this in our findings. NAC increases the TAC through the elevation of GSH and an increase in the activity of GPx, and this is associated with decreased MOF. There is a decrease in the SOFA score of 42% on the fifth day. NAC has antioxidant properties, increasing GSH levels and reducing the ROS, possibly leading to the inhibition of the effect of proinflammatory cytokines [64] and vasodilator activity on the microcirculation [32]. NAC also reduces the SOFA score and the LPO in patients with SARS-CoV-2 infection [29].

Other antioxidants such as MT could also be useful in patients with septic shock. We have found that it reduces the SOFA score and the LPO in SARS-CoV-2 infection. The reproducibility of the findings reinforces what was recently published in a pilot group, where evidence of its effect on reducing organ damage was found [65]. A recent narrative review emphasized the role of MT in the protection of the lung, kidney, liver, brain and vascular function during sepsis in animal studies. It suggested that MT may protect from multi-organ damage by attenuating OS, inflammation, apoptosis, autophagy and mitochondrial dysfunction [66].

In in vitro and in vivo studies, MT scavenges ROS, thus protecting cell membrane lipids, cytosol proteins, and nuclear and mitochondrial DNA. It preserves the permeability of the membrane, increasing its fluidity [48,67,68], and reduces hydroperoxide levels in mitochondria, restoring GSH homeostasis and mitochondrial function in organelles under OS [53]. It is also able to stimulate γ-glutamylcysteine synthase, and therefore, it can increase the intracellular synthesis of GSH [69,70]. Added to this, it restores functional mitochondrial activity that is depressed in some pathological situations, reducing O_2_^–^ consumption and protecting organs from excessive oxidative damage [71].

In this study, we found that in patients treated with MT, the Se levels were maintained, ecSOD was decreased, and TAC and GPx were increased. Therefore, it is feasible that the therapeutic effect on other enzymes such as the one observed in the decrease in ecSOD may be beneficial to control the OS in sepsis.

On the other hand, Se is used by GPx as cofactor and is decreased in septic shock [72]. The Se administration as selenious acid or selenite in intravenous loading doses reduces mortality, improving organ dysfunction and decreasing infections in critically ill septic patients. This was reported in a review and phase II clinical trials [73,74]. Low activity levels in selenoenzymes that are Se dependent such as the GPx and TrxR have been reported in diseases related to OS, sepsis [75], cardiovascular diseases [76] and cancer [77]. We found low levels of Se in patients with septic shock and MOF, which confirms its reduction in these serious conditions and although the levels did not increase with the antioxidant therapy, they were at least maintained. This result contrasts with the decreased levels found in untreated patients, in whom there were no changes towards an improvement in the SOFA score. The exact mechanism of how this process can be carried out is still not fully understood and more studies are required to clarify it. However, our results suggest that antioxidant therapy is capable of maintaining Se levels and should be included in the adjuvant therapy of these patients. Se could be used in specific clinical trials in which nutritional status, bone metabolism and severity could be included.

The possible benefits of therapy with trace elements such as Se, Cu, Zn and Mn in critically ill patients have been investigated without clear results. With the results obtained in this study, Se levels are maintained, which supports the role of this metal in the regulation of OS. Multiple studies have evaluated the effect of Se supplementation in critically ill patients. However, there are still controversies regarding the path of administration (enteral vs. parenteral), the dose (high vs. low), the use of loading doses, the selection of patients (septic vs. non-septic) and the supplementation of other antioxidants (mono therapy vs. cocktails), [73,76,77,78,79,80,81,82]. There is a history of therapeutic power in the control the OS deregulation and in the control of the inflammatory process [81]. However, the equilibrium threshold in the concentration in this trace element is very important, because an excess could lead to selenosis and contribute to inflammation in various disorders [83,84,85].

On the other hand, the study of thiols in this series of patients was included since thiols are molecules that contain a hydrogen sulfide group in the side chain (SH) which may act as an antioxidant by stabilizing and reducing the bridge between proteins caused by free radicals by accepting an unpaired electron. This process is also regulated by TxrR, another selenoenzyme which was decreased in these patients. However, the antioxidant therapy increased the activity of this enzyme. Our results suggest that the increase in thiols can participate with a synergic effect with the Vit E and decrease the OS damage mechanism on protein carbonylation in patients with septic shock. Vit E and thiols may act together preventing and blocking LPO, carbonylation and increasing TAC [85]. In agreement with this, Vit E increased the level of thiols, and this difference was statistically significant.

This study confirms the effect of antioxidants on the reduction of MOF measured by the SOFA score, which occurs from the second day of treatment on. Studies to follow should consider an adequate evaluation of the nutritional status and the participation of OS, and its consequences such as cytopathic hypoxia. This condition could explain the failure of different strategies used in the clinical management of septic shock, since when the mitochondrial machinery is blocked, many therapies result in unsuccessful efforts aimed at improving tissue oxygenation by increasing systemic oxygen supply and/or optimizing cardiovascular function.

Specific pharmacological treatments to modulate or block components of the inflammatory process have not achieved the expected success. The participation of OS in the pathways of damage in patients with septic shock constitutes a solid foundation to propose an adjuvant antioxidant therapy for sepsis and septic shock. This therapy may improve these conditions by regulating enzymatic and non-enzymatic pathways as previously observed in animal models. Studies are needed to substantiate the interaction and participation of nitrosative stress, OS and the interaction of cytokines in the pathogenesis of sepsis. Nevertheless, the antioxidant therapy modulates the over-synthesis of NO and nitrosative stress, reducing organic dysfunction.

These results raise new expectations for antioxidant treatment in MOF caused by sepsis. There are still gaps that need to be solved and these constitute areas of opportunity to explore in the future. The exact doses, the time of use and synergistic effects of combined use of several simultaneous antioxidants still remain unexplored.

## 5. Conclusions

The addition of antioxidant therapy to standard therapy in patients with septic shock decreases MOF and regulates the inflammatory state and the OS. Vit C therapy increases its serum levels and decreases CRP, PCT and NO_3_^−^/NO_2_^−^ levels. Vit C NAC and MT decrease SOFA score and LPO and improve TAC. Vit E increases thiol levels and tends to lower LPO. Treatment with Vit C, Vit E, NAC and MT maintains Se levels. The combined use of antioxidants in patients with septic shock is a perspective to be followed through randomized clinical trials, since their integral therapeutic target on OS pathways and their correlation with better clinical and pathophysiological outcomes could probably be demonstrated.

## Figures and Tables

**Table 1:** Demographic characteristics and comorbidities by treatment group at admission in ICU.

Characteristics	Vit C n = 25	Vit E n = 27	NAC n = 24	MT n = 26	Without Tx n = 29	*p*
Age, years Median (range)	62 (58–78)	70 (51–77)	69 (59–78)	62.5 (58–69)	75 (65–81)	0.10
IMC Median (range)	25 (23–30)	25 (22.8–29)	23 (21–26)	25.(21–28)	25 (23–28)	0.41
Men, n and (%) Women, n and (%)	10 (40) 15 (60)	17 (63) 10 (37)	14 (58) 10(42)	13 (50) 13 (50)	16 (55) 13 (45)	0.53 0.56
SAPS II, Mean ± SD	39.4 ± 14.1	45.7 ± 16.3	42.4 ± 19.84	40.8 ± 16.8	47.2 ± 7.1	0.60
APACHE II Median (range)	14 (12–19)	20 (15–24)	15.5 (11–21)	16.5 (10–21)	17 (15–25)	0.15
SOFA, Median (range)	8 (6–9)	9 (7–11)	8 (4–10)	8 (6–9)	9 (7–11)	0.42
NUTRIC, Mean ± SD	4.1 ± 2.2	4.8 ± 1.6	4.0 ± 1.8	3.8 ± 1.7	5.1 ± 1.5	0.41
Diabetes Mellitus, n and (%)	7 (28)	5 (19)	5 (21)	6 (23)	8 (28)	0.90
Arterial hypertension, n and (%)	10 (40)	11 (41)	12 (50)	8 (31)	15 (52)	0.53
COPD, n and (%)	1 (4)	5 (19)	4 (17)	2 (8)	0 (0)	0.05
Smoking, n and (%)	17 (68)	12 (44)	9 (38)	15 (58)	14 (48)	0.22
Cancer, n and (%)	6(24)	11 (41)	8 (33)	8 (31)	14 (48)	0.39
Cirrhosis, n and (%)	2 (8)	2 (7)	1 (4)	1 (4)	4 (14)	0.71
Chronic kidney disease, n and (%)	2 (8)	3 (11)	4 (17)	3 (12)	3 (10)	0.92
Hypothyroidism, n and (%)	4 (16)	4 (15)	2 (8)	6 (23)	7 (24)	0.56
CVD, n and (%)	3 (12)	0 (0.00)	1 (4)	2 (8)	3 (10)	0.41
AMI, n and (%)	1 (4)	0 (0)	3 (13)	2 (8)	2 (7)	0.43
Atrial fibrillation, n and (%)	3 (12)	2 (7)	3 (13)	5 (19)	4 (14)	0.79
Pulmonary and CNS	0 (0)	0 (0)	1 (4)	0 (0)	0 (0)	0.20

Abbreviations: Vit C = vitamin C, Vit E = vitamin E, NAC = N-acetylcysteine, MT = melatonin, Tx = without treatment, COPD = chronic obstructive pulmonary disease, CVD = cerebral vascular disease, AMI = acute myocardial infarction, CNS = central nervous system, SAPS II = Simplified Acute Physiology Score, APACHE II = Acute Physiology and Chronic Health Evaluation, SOFA = sequential organ failure assessment, NUTRIC: Nutrition Risk in the Critical III.

**Table 2:** Condition of the patients at the time of admission, according to the assigned antioxidant therapeutic management and the type of standard management and ventilatory assistance.

Admission to the ICU	Vit C n = 25	Vit E n = 27	NAC n = 24	MT n = 26	Without Tx n = 29	*p*
Reason for admission n (%)						
Septic shock from surgery	7 (28)	6 (22)	5 (21)	4 (15	13 (14)	0.14
Septic shock from non-surgical	18 (72)	21 (78)	19 (79)	22 (84)	16 (55)	
Infection site n (%)						
Pulmonary	7 (29)	11 (41)	9 (39)	11 (42)	10 (34)	0.83
Gastrointestinal	10 (42)	8 (30)	5 (22)	5 (19)	11 (38)	
Nephro-Urinary	3 (13)	3 (11)	6 (26)	6 (23)	3 (10)	
CNS	0 (0)	2 (7)	0 (0)	0 (0)	1 (3)	
Skin and soft tissues	2 (8)	2 (7)	2 (9)	2 (8)	2 (7)	
Endocarditis	0 (0)	0 (0)	0 (0)	0 (0)	1 (3)	
Gastrointestinal-Urinary	0 (0)	1 (4)	0 (0)	2 (8)	1 (3)	
Pulmonary-CNS	0 (0)	0 (0)	1 (4)	0 (0)	0 (0)	
Pulmonary-Gastrointestinal	1 (4)	0 (0)	0 (0)	0 (0)	0 (0)	
Variables median (min-max ranges)						
Temperature	36 (36–37)	36 (36–37)	36 (36–37)	37 (36–37)	36 (36–37)	0.34
Cardiac Frequency	83 (68–94)	85 (66–108)	86 (66–97)	86 (67–100)	83 (74–104)	0.98
PVC, mmHg	11.5 (6.5–14)	9 (6–12)	12 (7–13)	9 (7–13)	13 (9–17)	0.15
MAP, mmHg	73 (63–83)	77 (73–81)	78 (68.5–85)	73 (65–81)	78 (65–82)	0.52
Minime MAP, mmHg	63 (54–72)	68 (61–75)	63 (55–68)	61 (55–72)	61 (54–68)	0.11
PaO_2_/FiO_2_, mmHg	196 (129–309)	240 (120–266)	239 (141–300)	197 (115–242)	190 (150–250	0.93
Lactate, mmol/L	1.3 (1.09–2.4)	2.4 (1.4–3.7)	2.0 (1.4–3.2)	2.3 (1.49–4.6)	2.6 (2.08–4.05)	0.01
Bilirubins mg/dL	0.8 (0.42–1.1)	1.2 (0.6–2.2)	0.9 (0.46–1.9)	1.02 (0.5–1.8)	1.1 (0.7–2.3)	0.27
Hemoglobin g/dL	12 (10–14)	11 (10–15)	11 (10–11)	11 (10–13)	12 (10–14)	0.21
Leucocytes 10^3^/µL	11 (8–19)	10 (7–16)	9 (8–14)	13 (10–20)	14 (7–21)	0.35
Procalcitonin ng/dI	1.2 (0.5–30)	4 (0.5–40)	1.2 (0.3–10)	2.8 (1.04–8.4)	7.6 (1.4–32)	0.33
C Reactive protein mg/dI	20 (11–31)	25 (12–32)	12 (8–23)	22 (10–29()	23 (11–33)	0.38
Platelets	241 (186–295)	166 (82–258)	161 (110–215)	245 (161–443)	212 (131–297)	0.01
Inotropic type (n and %)						
Steroid treatment	6 (24.00)	16 (59.26)	9 (37.50)	8 (30.77)	14 (48.28)	0.07
Enteral, nutrition	21 (87.50)	23 (85.19)	21 (87.50)	24 (96.00)	23 (79.31)	0.50
Parenteral nutrition	4 (16.67)	7 (25.93)	7 (29.17)	5 (20.00)	9 (31.03)	0.73
Inotropic	2 (8.33)	7 (25.93)	3 (12.50)	5 (19.23)	10 (34.48)	0.14
Dobutamine	1 (4.17)	1 (3.70)	0 (0.00)	0 (0.00)	1 (3.45)	
Levosimendan	0 (0.00)	6 (22.22)	2 (8.33)	4 (15.38)	8 (27.59)	0.06
Dopamine	1 (4.17)	0 (0.00)	0 (0.00)	1 (3.85)	0 (0.00)	
Vasopressor type (n and %)						
Norepinephrine Vasopressin, Norepinephrine + Vasopressin,	16 (67) 0 (0) 8 (33)	11 (41) 1 (4) 15 (56)	16 (67) 0 (0) 16 (70)	13 (50) 0 (0) 16 (62)	17 (59) 0 (0) 23 (79)	0.26
Other treatments						
Antifungal	6 (25.00)	6 (22.22)	7 (29.17)	6 (23.08)	11 (37.93)	0.70
RTT	1 (4.17)	3 (11.11)	2 (8.33)	1 (3.85)	3 (10.34)	0.80
Mechanic ventilation	13 (54.17)	15 (55.56)	16 (69.57)	16 (61.54)	23 (79.31)	0.26
IMV	12 (50) 1 (4)	12 (44) 4 (15)	11 (48) 5(22)	10 (38) 5(19)	19 (66) 2 (7)	0.39

Abbreviations: Vit C = vitamin C, Vit E = vitamin E, NAC = N-acetylcysteine, MT = melatonin, Tx = COPD = chronic obstructive pulmonary disease, CVD = cerebral vascular disease, PVC = peripheral venous catheters, AMI = acute myocardial infarction, CNS = central nervous system, IMV = intermittent mandatory ventilation, SAPS II = Simplified Acute Physiology Score, APACHE II = acute physiology and chronic health evaluation, SOFA = sequential organ failure assessment, MAP = mean arterial pressure, RTT = referral to treatment. Treatment statistics, Kruskal–Wallis and Fisher. The values are expressed as median (Min-Max).

## Data Availability

The data in our study are available from the corresponding author upon reasonable request.
